# Erectile Dysfunction in Young Adults: A Narrative Review

**DOI:** 10.7759/cureus.89918

**Published:** 2025-08-12

**Authors:** Ali Safa, Chady Waked

**Affiliations:** 1 Urology, Hôtel-Dieu de France, Beirut, LBN; 2 Faculty of Medicine, Université Saint-Joseph de Beyrouth, Beirut, LBN; 3 Urology, Université Saint-Joseph de Beyrouth, Beirut, LBN

**Keywords:** erectile dysfunction, male sexual health, organic erectile dysfunction, psychogenic erectile dysfunction, young adult male

## Abstract

Erectile dysfunction (ED), traditionally considered a condition of older men, is increasingly reported among men under the age of 40, yet its prevalence in this population remains underestimated. While psychogenic factors have been considered the primary cause, ED is now recognized as a multidimensional condition with both organic and psychological aspects. Hence, this review aims to synthesize current evidence on the epidemiology, psychosocial impact, etiological factors, and management strategies of ED in the young male population. A narrative review was conducted using PubMed’s search builder, applying a combination of the following Medical Subject Headings and keywords: “Erectile Dysfunction” and “Young.” The search identified 1,981 articles, from which articles were selected based on relevance and methodological quality. The prevalence of ED in young men varies widely, with studies reporting rates as high as 35%. While psychogenic causes such as performance anxiety, depression, and relationship issues are prevalent, a significant proportion of young men also present with organic etiologies, including endothelial dysfunction, hormonal imbalances, metabolic syndrome, and neurogenic disorders. Lifestyle factors such as pornography use, poor sleep quality, dietary habits, lack of physical activity, and recreational drug use have also been independently linked with ED. Management of ED in this population requires a multidisciplinary approach that incorporates both pharmacologic treatments (e.g., phosphodiesterase 5 inhibitors, hormone therapy) and non-pharmacologic interventions, including lifestyle modification and cognitive-behavioral therapy. ED in young men is an increasingly prevalent and clinically complex condition that necessitates comprehensive evaluation and individualized treatment. Further research is essential to improve outcomes in this growing patient population.

## Introduction and background

Erectile dysfunction (ED) is characterized by a recurrent or persistent inability, whether partial or complete, to attain or sustain an erection sufficient for satisfactory sexual intercourse, despite appropriate erotic stimulation. This term has replaced the earlier designation of “impotence” [1].

ED can result from a variety of factors, mainly categorized into organic, psychogenic, and lifestyle or drug-related causes [2]. Organic factors include conditions such as cardiovascular disease, diabetes, hormonal imbalances (such as hypogonadism), neurologic conditions, and iatrogenic etiologies [3]. Psychogenic causes often involve psychological conditions such as anxiety, depression, stress, past sexual trauma, and relationship difficulties [4]. Furthermore, lifestyle behaviors such as smoking, excessive alcohol consumption, physical inactivity, and obesity, as well as the use of certain medications (e.g., antidepressants, antihypertensives, antipsychotics) and recreational drugs, have also been associated with ED [5].

The diagnosis of ED involves a comprehensive evaluation to determine the underlying causes and guide appropriate treatment. The medical history, including sexual history and medication use, is crucial during the initial basic workup of ED [5]. Many questionnaires have been developed to assess ED, with the International Index of Erectile Function being one of the most commonly used [6]. In addition, it is also essential to include questionnaires that explore the psychogenic factors during the initial assessment, as psychological components often contribute significantly to the condition [2]. Physical and andrological examination includes assessment of vital signs, examination of secondary male characteristics (e.g., gynecomastia, body hair), body mass index (BMI), scars from previous interventions and traumatism, and genital assessment of penis and testis (e.g., meatus position, penis scars or lesions, Peyronie’s disease, testis size) [7]. Laboratory tests are performed to screen for diabetes (HbA1c), hyperlipidemia, and hormonal testing for hypogonadism (testosterone, prolactin, thyroid hormones) [3]. Penile ultrasound and nocturnal penile tumescence test are also available options, despite not being used in routine assessment [8].

Available treatment options for ED include oral phosphodiesterase type 5 inhibitors (PDE5i) as the first-line therapy, followed by intracavernosal injections, hormone replacement therapy, vacuum erection devices, and penile prosthesis implantation for severe or refractory cases [2,3]. In recent years, emerging therapies such as low-intensity extracorporeal shockwave therapy, stem cell injections, and platelet-rich plasma therapy have gained attention and are currently under clinical investigation for their potential to restore erectile function through tissue regeneration [9].

Although ED has traditionally been regarded as an age-related condition, recent studies report increasing prevalence among younger men [10]. In the last decade, advancements in social media, increased use of technology, evolving lifestyle habits among youth, alongside the impact of the COVID-19 pandemic have directly affected sexual health and behaviors. Some reports have shown a rise in the prevalence of ED in young men, with certain studies reporting up to a 31-fold increase since 2014 [11]. Despite this, the topic remains underrepresented in the literature, which highlights the importance of addressing it in this review. Therefore, this review aims to explore the psychosocial impact, prevalence, evaluation, causes, and management of ED in young males under the age of 40.

## Review

Methodology

This narrative review was conducted to explore the epidemiology, etiology, psychosocial impact, and management of ED in young males. A comprehensive literature search was performed in PubMed using a combination of Medical Subject Headings and keywords, including “Erectile Dysfunction” and “young.” The search time frame included all articles published up to 2025, with no lower date restriction.

The initial search yielded 1,981 articles. Articles were screened in two stages: first, titles and abstracts were reviewed for relevance to ED in young men. Second, full texts of potentially relevant articles were assessed. Inclusion criteria were original research studies, reviews, and meta-analyses focused specifically on ED in young males, specifically under 40 years of age. Exclusion criteria included case reports with limited patient numbers, studies without clear methodology, and articles not written in the English language. Duplicates were identified and removed. Irrelevant articles were excluded based on the screening criteria.

Given the narrative nature of this review, a formal quality appraisal or risk of bias assessment was not conducted. However, studies were selected based on relevance and methodological clarity to ensure the inclusion of high-quality evidence. Data from the included articles were synthesized thematically, focusing on epidemiology, impact, etiologies (psychogenic and organic), and management of ED in young males.

Epidemiology

Multiple reports indicate a rising incidence of ED among men under the age of 40 [12]. In a U.S. cohort study involving 2,660 young men, the overall prevalence of ED was 14.2%, with 11.3% experiencing mild ED and 2.9% reporting moderate-to-severe symptoms [13]. Other multinational studies have demonstrated that the prevalence of ED in men younger than 40 is often underestimated and may be as high as 35% [14]. Although psychogenic ED is often considered the primary cause in younger individuals, a thorough evaluation of organic factors should not be overlooked, as ED is now recognized as a multifactorial condition [15,16]. A Turkish study examining ED across different age groups found that 14.8% of men under the age of 40 had an identifiable organic cause for their condition [17]. In addition, another study conducted on a smaller population reported that psychogenic ED was diagnosed in 50% of the patients, while 45% were found to have an organic cause [18].

Furthermore, data on young homosexual populations have indicated a higher incidence of ED compared to the heterosexual population. An Italian meta-analysis reported that homosexual orientation was associated with a 1.5-fold increase in the odds of experiencing ED [19]. Similarly, a North American study conducted among medical students found a higher prevalence of ED among those identifying as homosexual [20]. Consistent findings were reported in a Peruvian study conducted in a young homosexual population, where multiple sexual partners and shorter relationship duration were identified as factors more frequently associated with ED [21].

Psychosocial Impact of ED in the Young Population

Sexual activity has an important impact on men’s lives; many reports have found an association between ED and low self-esteem [22]. A Brazilian study conducted on a population of 1,947 men aged between 18 and 40 years has shown that ED caused a negative impact on recreational activities, work, relationships, and sexual life satisfaction [14]. Thus, a prompt evaluation of mental health status within this population is essential for effective treatment and management of young patients suffering from ED [21].

Etiologies

Pornography Consumption and ED: A Myth or a Reality?

Today, easy access to the internet has significantly increased the consumption of pornographic content, particularly among young adults. In recent years, concerns have been growing regarding a potential association between frequent pornography use, regular masturbation, and the development of ED [23]. A study conducted in Belgium investigating the association between ED and the degree of pornography addiction in a population of men aged between 18 and 35 years found that higher levels of pornography consumption were linked to increased rates of ED (p < 0.001). In contrast, the frequency of masturbation was not associated with a higher prevalence of ED (p = 0.17) [24]. However, another study conducted by Rowland et al. on a population of men aged under 30 did not find any association between pornography consumption and ED or ED severity (p = 0.79) [25]. Therefore, further research is needed to explore this association and identify the factors that may mediate or influence the relationship between pornography consumption and sexual dysfunction.

Psychogenic ED

It is generally reported that patients suffering from psychogenic ED experience a sudden onset of symptoms, with decreased libido while maintaining good quality of spontaneous or self-stimulated erections [10]. Manalo et al. found a significant association between ED and symptoms of depression and anxiety in men aged 18 to 40 years who were already diagnosed with ED (p < 0.001) [26]. This suggests that anxiety and depression may often accompany ED after its onset. However, other studies indicate that this relationship is likely bidirectional, as individuals with pre-existing depression or anxiety are also at higher risk of developing ED [27]. A study conducted among 3,975 participants concluded that the deterioration in couples’ relationships was strongly correlated with sexual dysfunction such as ED (p = 0.018) and resulted in higher rates of depression and anxiety [28]. Other reports have found the same association with low relationship satisfaction (p = 0.04) and low sexual interest among the young population (p < 0.001) [25]. Proposed theories regarding the pathophysiology of psychogenic ED suggest that elevated stress levels may lead to increased cortisol secretion, which, in turn, contributes to vasculogenic resistance, resulting in ED among adolescent and young adult populations [11]. The physician’s evaluation of the psychogenic aspects of ED is often challenging due to the complex approach required, which involves consideration of the patient’s partner and incorporates social, interpersonal, and lifestyle-related factors [29].

Organic ED

Organic ED has often been considered a predictive factor for future comorbidities even among the young population suffering from ED [18]. Generally, organic etiologies in the young population include vasculogenic, neurogenic causes, Peyronie’s disease, adverse effects of medications, and endocrine disorders [15].

Vasculogenic ED is primarily attributed to two mechanisms, namely, focal arterial occlusion and endothelial dysfunction [30]. Sommer et al. have found that cycling-induced arterial occlusion due to seat perineal compression was significantly associated with ED [31]. In addition, a study conducted on 122 patients under 40 reported that patients suffering from ED presented higher levels of systolic blood pressure, total cholesterol and triglyceride, high sensitivity C-reactive protein, greater carotid intima-media thickness, and Framingham risk score, reflecting subclinical endothelial dysfunction [32].

Many reports have shown an association between ED and diabetes in the young population. A study including patients between 18 and 40 years has shown a positive correlation between ED and patients with pre-diabetes or type 2 diabetes (RR = 1.34; 95% CI = 1.16-1.55) [33]. The same result was found in a population of patients aged between 18 and 35 years, where ED had a higher prevalence in patients suffering from type 1 diabetes (p < 0.001) [34].

Obesity has consistently been associated with ED. Reports among young men have shown that a higher BMI and a greater number of metabolic syndrome components, such as unfavorable body composition, dyslipidemia, and insulin resistance, are more prevalent in individuals with ED. A study conducted in Spain on 254 young, non-diabetic men reported a prevalence of ED in 42.1% of participants [35,36].

A retrospective study involving 2,292 men under the age of 40 with ED reported that 10.7% of patients exhibited low testosterone levels [37]. Identified etiologies of hypogonadism include primary forms such as Klinefelter’s syndrome and cryptorchidism, as well as secondary (hypogonadotropic) causes, including congenital conditions such as Kallmann syndrome and acquired disorders such as prolactinomas and traumatic brain injury [15]. In addition, a study conducted in China found that, among eugonadal young men, a higher estradiol-to-testosterone ratio was associated with the presence of ED, particularly in cases of vascular organic ED [38].

Among neurogenic causes of ED in the young population, multiple sclerosis has been identified as a significant risk factor for ED (p < 0.001) [39]. Furthermore, a study involving 80 men with epilepsy, aged 22 to 50 years and without other identifiable causes of ED, demonstrated a positive association between epilepsy and ED [40]. The use of antiepileptic drugs and neuroleptics has also been linked to ED [41,42]. Additionally, recreational drugs such as ketamine, cocaine, and cannabis are associated with ED, particularly among younger individuals, where the prevalence of such use is increasing [43,44]. Moreover, certain medications commonly prescribed in the young population, such as 5-alpha-reductase inhibitors used for the treatment of androgenetic alopecia, have also been implicated in the development of ED (p < 0.001) [45].

A history of surgery has also been associated with an increased risk of ED [5]. A study conducted in Taiwan demonstrated that patients aged 15 to 45 years who sustained pelvic fractures and underwent angioembolization were more likely to develop ED (OR = 32.637; 95% CI = 14.137-75.346; p < 0.001) [46]. Similarly, an elevated incidence of ED was observed in men under 50 years of age who underwent surgery for lumbar spine disorders, despite the absence of identifiable pre-existing risk factors for ED (p = 0.03) [47]. Peyronie’s disease has also been associated with ED in the young population. A study conducted in Turkey reported that ED was significantly correlated with Peyronie’s disease, with 21% of affected patients presenting with ED [48].

The COVID-19 pandemic had an impact on nearly every aspect of daily life, including sexual health. The pandemic not only disrupted routines and social interactions but also affected sexual behavior and contributed to an increase in reported cases of ED [49].

In fact, one major factor was the widespread change in lifestyle during lockdowns. Increased isolation, reduced face-to-face interaction, and a greater reliance on virtual communication all contributed to a decline in real-world intimacy. As people spent more time alone and online, often engaging with social media or digital forms of connection, many found themselves drawn into virtual relationships that lacked the emotional and physical depth of in-person interactions. For example, a study conducted by Zattoni et al. showed a significant increase in the use of pornographic websites during the COVID-19 pandemic [50].

Erectile function has been affected through multiple pathways during the COVID-19 pandemic. First, COVID-19 itself has been associated with organic ED due to its impact on the vascular system, particularly endothelial dysfunction. Second, there has been a notable rise in psychogenic ED, driven by elevated levels of stress, anxiety, disrupted routines, and decreased physical and social activity [51].

Management

The management of ED in young men presents a clinical challenge. As with all patients presenting with ED, it is essential to identify the underlying etiology before initiating treatment [52]. Lifestyle modifications may play a particularly important role in the management of ED in this population [15]. A Peruvian study demonstrated an independent and significant association between ED and poor sleep quality [53]. Similarly, dietary factors appear to contribute to ED; one study found that young men with ED had a lower intake of flavonoid-rich foods [54]. Additional lifestyle factors such as weight loss, smoking cessation, regular physical activity, and reduced alcohol consumption are well-established in improving erectile function [15,55,56].

Conventional treatment options for ED remain viable in younger individuals. These include PDE5i, hormone replacement therapy in cases of confirmed hypogonadism, intracavernosal injections, and penile prostheses. Moreover, cognitive-behavioral sex therapy has shown outcomes comparable to those of sildenafil 50 mg in the treatment of non-organic ED [57].

Response to Treatment in Young Men

The treatment of ED in young adults is similar in approach to that in older adults, but responses can differ significantly due to differences in underlying causes, psychological factors, and lifestyle elements [52]. In fact, a comparison between younger and older patient groups demonstrated that the younger cohort exhibited a more favorable response to various treatment modalities [58]. This may be attributed to better overall health status, fewer comorbidities, and preserved endothelial function in younger individuals [52,58]. However, several factors may influence the response to treatment. For example, a study conducted in China among young adults found that a history of alcohol consumption, lack of spousal cooperation, multiple fixed sexual partners, longer duration of ED, and the presence of depression were all associated with non-responsiveness to PDE5i therapy [59]. In addition, the recreational use of ED treatments, such as PDE5i and intracavernosal injections, has been increasingly reported among young adults. This trend underscores the importance of adhering strictly to evidence-based clinical guidelines for the management of ED to prevent inappropriate prescribing practices and reduce the risk of drug-related adverse effects [60].

Discussion

Despite the predominance of psychogenic factors, our study emphasizes that organic causes play a substantial role in ED among young men. These findings are especially important given the role of ED as an early clinical marker of systemic diseases, including diabetes and cardiovascular conditions, even in asymptomatic individuals [61]. For example, a study by Yao et al. demonstrated that ED in young men may represent the first clinical manifestation of insulin resistance [62]. Therefore, early identification of ED in young men may offer a valuable opportunity for the detection and management of otherwise silent systemic pathologies.

The psychosocial dimensions of ED in young populations are particularly complex. The evidence strongly supports a bidirectional relationship between ED and mental health conditions [63]. In addition, concerns around body image, sexual performance, and societal pressures contribute to performance anxiety and sexual dissatisfaction [64]. These elements suggest that a biopsychosocial approach is vital for effective evaluation and management of ED in younger men. Unfortunately, stigma often prevents this group from seeking medical attention, leading to delays in diagnosis and intervention [65].

The possible link between pornography use and ED reflects the changing sexual habits of today’s youth. Some studies suggest that excessive porn use may be related to ED, but the evidence is still unclear. More research is needed to find out whether this connection is causal or just a coincidence, and whether other factors, for example, unrealistic sexual expectations, might be involved.

As previously discussed, ED in young men often results from a combination of organic and psychogenic factors, necessitating an individualized treatment approach. Behavioral therapy should be considered part of a comprehensive management plan. Furthermore, lifestyle modifications have the potential to improve erectile function and contribute to favorable effects on cardiometabolic and endothelial health, particularly in individuals with metabolic syndrome.

To enhance understanding and improve outcomes in this population, future research should focus on exploring the long-term consequences of ED in young men, including its impact on quality of life and overall health. In addition, well-designed prospective longitudinal studies are needed to clarify causal mechanisms and track the progression of associated risk factors. Finally, there is a need to develop and validate age-appropriate diagnostic tools and treatment strategies that reflect the psychosocial and clinical characteristics of younger patients.

## Conclusions

ED in young men is an increasingly prevalent condition and is no longer considered age-related. It is now recognized as a multidimensional disorder with a wide range of contributing psychogenic and organic factors. Accurate identification of the underlying etiology is essential to guide effective and individualized treatment strategies. In addition to conventional therapeutic approaches, lifestyle modification plays a critical role in the management of ED in younger individuals. Further research is needed to fully explain the complex mechanisms and risk factors associated with ED in this population.
